# Feasibility and patency of echoendoscopic anastomoses with lumen apposing metal stents depending on the gastrointestinal segment involved

**DOI:** 10.1038/s41598-021-83618-x

**Published:** 2021-02-17

**Authors:** Maite Betés, Pablo Pérez-Longo, Sandra Peralta, Alejandro Bojorquez, Ramon Angós, Ana Chopitea, Jorge Baixauli, Miguel Munoz-Navas, Jose Carlos Súbtil

**Affiliations:** 1 Department of Gastroenterology, Endoscopy Unit, University of Navarra Clinic, Pamplona, Spain; 2 Department of Gastroenterology, University of Navarra Clinic, Pamplona, Spain; 3 Department of Medical Oncology, University of Navarra Clinic, Pamplona, Spain; 4 Department of General Surgery, University of Navarra Clinic, Pamplona, Spain

**Keywords:** Gastroenterology, Oncology

## Abstract

EUS-guided anastomoses with LAMS have emerged as a therapeutic option for patients with obstruction of the digestive tract. However, the long-term permeability of these anastomoses remains unknown. Most of the published cases involve the gastric wall and experience in distal obstruction is limited to few case reports. We review our series of patients treated with LAMS for gastrointestinal obstruction and describe the technical success according to the anastomotic site and the long-term follow-up in those cases in which the stent migrated spontaneously or was removed. Out of 30 cases treated with LAMS, EUS-guided anastomosis did not involve the gastric wall in 6 patients. These procedures were technically more challenging as two failures were recorded (2/6, 33%) while technical success was achieved in 100% of the cases in which the stent was placed through the gastric wall. In two of the patients, one with entero-enteric and another with recto-colic anastomosis, stent removal after spontaneous displacement was followed by long term permeability of the EUS-guided anastomosis (172 and 234 days respectively). In a EUS-guided gastroenterostomy the stent was removed at 118 days, but closure of the fistula was confirmed 26 days later. Our experience suggests that LAMS placement between bowel loops is feasible and might allow the creation of an anastomosis with long-term patency. As compared to LAMS placement between bowel loops, when LAMS are placed through the gastric wall, removal of the LAMS seems to lead to closure of the fistula.

## Introduction

The lumen-apposing metal stent (LAMS) was originally designed for transmural drainage of pancreatic fluid collections and in the last few years, it has been used extensively for that indication. Its extensive use has led to other in-and off-label indications to be proposed^1^. In 2012, the use of a LAMS for EUS-guided gastroenterostomy (EUS-GE) in a pig model was reported^2^. The stent anchors, designed to prevent detachment, distribute pressure evenly on the luminal wall and securely hold the jejunal or duodenal wall to the gastric wall. A proprietary silicone-based material fully covers the stent, in order to prevent tissue ingrowth and facilitate removal.

In the clinical setting, EUS-GE have been used as a bridge to definitive treatment in benign gastric outlet obstruction (GOO)^3,4^, or as a palliative approach in patients with advanced malignant disease ^5–7^. The key point for stent release is to find a suitable position between the proximal and the distal lumen that is close enough and without interposed vessels. Although the anastomotic site could theoretically be found in any part of the gastrointestinal tract, most of the reported cases involve the gastric wall and the small intestine, and experience in distal obstruction is limited to few case reports ^8,9^.

When used for benign GOO, authors recommend removing the stent as soon as there is evidence that the primary obstruction has resolved^4^. This recommendation for removal does not apply to cases of malignant obstruction where LAMS can serve as palliative therapy. To our knowledge, there is no published data about the long-term permeability of the fistula created upon the removal of the LAMS or after its spontaneous migration.

Since March 2018, we have performed 30 EUS-guided anastomoses with LAMS. Our experience includes anastomoses with the stomach and more distal anastomosis. Herein we describe the largest series of patients treated with LAMS outside the gastric wall published to date. We also report the long-term follow-up of the fistulous tracts after spontaneous migration or removal of the stent and discuss the possible future applications these findings.

## Patients and methods

We retrospectively reviewed all the cases of EUS-guided anastomoses (EUS-A) with LAMS performed in our center from March 2018 to October 2019, describing the technical success according to the anastomotic site and long-term follow-up in those cases in which the stent was removed or migrated spontaneously.

The patients included were consecutive cases in whom, after a multidisciplinary assessment, endoscopic LAMS placement was considered appropriate. Treatment options were discussed on an individual basis. All patients provided written informed consent.

All methods were carried out in accordance with relevant guidelines and regulations. For this retrospective case series, we obtained approval from the research ethics committee of the University of Navarre.

One experienced therapeutic endoscopist performed all procedures, with or without trainee involvement. The AXIOS-ECTM stent (“Hot Axios”, 20-mm diameter; Boston Scientific, Galway, Ireland) was used in all cases. It includes an electrocautery enhanced delivery system which allows puncture and release of the stent in a single-step procedure, thus reducing the number of accessories to be exchanged and potentially decreasing the complication rates^10^.

To identify the targeted intestinal loop, the luminal water filling technique^10^ was used in most cases, passing a naso-biliary drainage catheter through the stenotic segment with the help of endoscopes of different diameters. When the targeted loop was pre-stenotic and distended, water instillation was not necessary. All cases were performed with a therapeutic linear echoendoscope (GF-UCT180; Olympus, Hamburg, Germany). Freehand direct access and intra-channel release technique was used in all cases. Dilation of the lumen apposing metal stent was not performed in any case.

All patients underwent general anesthesia, and endotracheal intubation was only used in patients with per-oral approach.

Technical success was defined as adequate positioning and deployment of the stent as determined endoscopically and radiographically.

Patients remained hospitalized and initiated a liquid diet immediately after or up to 2 days later depending of the type of procedure. The patients were discharged home when they showed adequate tolerance to diet progression.

## Results

Thirty patients were included in this retrospective study, of whom 16 were male (53.3%). Mean age was 67.1 $\pm$ 11.54 years (range 34–90). Median follow-up was 164 days (IQR 48–289). The indication for EUS-A was a malignant obstruction in 27 patients, 22 of whom had distant metastases. In 24 patients the EUS-A was the first-choice technique, while in 6 it was a rescue procedure after a failed endoscopic (2 cases) or surgical (4 cases) approach.

Twenty-four (80%) LAMS had their proximal flange located in the gastric lumen, whereas in 6 (20%) the gastric wall was not involved. EUS-guided anastomosis without gastric wall involvement are described in Tables 1 and 2.

**Table 1:** Characteristics of patients undergoing EUS-guided anastomosis without involvement of the gastric wall.

	Age (ys)	Sex	Site of obstruction	Site of LAMS placement	Surgically altered anatomy	Technical success	Adverse events
Case 1*	65	Female	Colo-rectal anastomosis	Colo-rectal	Yes Rectal stump. Diverting ileostomy	Yes	No
Case 2*	57	Male	Jejunum	Jejunal-jejunal	Yes Surgical gastroenteroanastomosis	Yes	Displacement (at 3 mo)
Case 3	35	Female	Jejunum ALS^1^	Jejunal-jejunal	Yes Gastrectomy^2^, Roux-en-Y hepático-jejunostomy	Yes	No
Case 4	61	Male	Ileum (pelvic tumoral implant)	Ileo-sigmoid	No	No^3^	Target loop perforation (resolved with endoscopic clips)
Case 5	58	Female	Ileum	Ileo-ileal	Yes ARR^4^	Yes	Leakage at the LAMS site (surgery not required)
Case 6	57	Male	Pelvic ileum	Ileo-colonic	Yes ARR^4^ + diverting ileostomy	No	Perforation (resolved surgically)

1. Afferent loop syndrome.

2. Patient with diffuse gastric cancer.

3. The goal was to create an ileo-sigmoidostomy. Although the LAMS was released without incident, the punctured target loop turned out to be the jejunum instead of the ileum. Therefore, a jejuno-sigmoidostomy was created.

4. *ARR* Anterior resection of the rectum.

*These are the two patients described in the text, in which the long-term permeability of the anastomosis was confirmed once the LAMS was removed.

**Table 2:** Follow-up of patients undergoing EUS-guided anastomosis without involvement of the gastric wall.

	Follow-up (months)	Recurrence requiring reintervention	Discharged to home	Stent dwell time (months)	Time to death (months)	Cause of death
Case 1*	15	No	Yes	15	Alive	––
Case 2*	9	No	Yes	9	9	Progression (carcinomatosis)
Case 3	1.5	No	Yes	1.5	1.5	Progression (carcinomatosis)
Case 4	2	Yes (laparoscopic bypass at 1 mo)	Yes	2	2	Progression (carcinomatosis)
Case 5	2	No	Yes	2	2	Progression (carcinomatosis)
Case 6	––	––	––	––	––	––

Technical success was achieved in 100% of transgastric procedures (Table 3), and all 24 patients proceeded to an oral diet. Of 21 cases with malignant GOO, we had clinical follow-up in 17 patients, and 16 of them died maintaining the oral diet 8–423 days after LAMS placement. Only one patient (described below) required surgical rescue after intentional stent removal, due to closure of the fistula. She is currently alive. 3 patients presented benign GOO, due to acute pancreatitis. Oral intake began the day after the LAMS procedure, with good tolerance. One patient required a second stent 25 days after the first procedure, due to GOO symptoms secondary to buried stent. A second overlapping LAMS was placed, and both were removed 171 days later, when there was evidence that the GOO had resolved; closure of the fistula was endoscopically confirmed 39 days later. The Axios stent has recently been removed in the other two patients after 513 and 625 days respectively and we are waiting for further follow-up.

**Table 3:** Characteristics and outcomes of EUS gastroenterosomies.

N	Clinical success	Malignant or Benign GOO	Diet at last follow-up	Etiology	Previous procedures	Adverse events (AE)	Reintervention for GOO symptoms	Follow-up (days, median)	Status
30	30	Malignant 21	Oral diet N = 21	Pancreatic cancer 11	Surgical 1			79 (8–223)	Death 10 Lost 1
				Duodenal cancer 2		1 Bleeding needing hemostatic therapy (mild AE)	Yes (1*)	1) 133 2) *	Death 1 Alive 1
				Gallbladder cancer 1				32	Death
				Gastric cancer 4	Endoscopic stent 1			263 (29–286)	Death 3 Lost 1
				Other 3				137 (70–423)	Death 1 Lost 2

		Benign 3						Stent removal (days)	Endoscopy after stent removal (days)
				Acute Pancreatitis 3			1 (second axios placed)	25 (1^st^ axios) 171 (2^nd^ axios)	39 (closed)
								513	–
								625	–

Other: pylorus, ectopic pancreas, lymphoma.

*Correspond to the last patient described in results: intentional removal of the stent led to the closure of the fistula and a surgical gastroenteroanastomosis was required. She is currently alive.

Of 6 patients in whom a lower EUS-A was intended, 2 failures were recorded. The only deployment failure (1/6, 16,7%) corresponded to an ileocolic anastomosis in a patient with stage IV pancreatic adenocarcinoma and a poor bowel preparation (Tables 1, 2, case 6). In addition, due to the great torquing maneuvers needed to reach the obstruction site, the Axios device failed to run fast enough and did not enter the ileal loop that contracted due to the electric shock. There was a perforation that required bowel resection and surgical anastomosis. The second case (Tables 1, 2, case 4), was a failed attempt to create an ileo-sigmoidostomy. Although the LAMS was released without incident, the punctured target loop turned out to be the jejunum instead of the ileum. Therefore, a jejuno-sigmoidostomy was created, and laparoscopic surgical rescue was needed 1 month later. In the other 4 patients (67%) we placed the LAMS successfully and without major complications. These cases include the following anastomoses (Tables 1, 2): colo-rectal (case 1), jejunojejunal (cases 2 and 3) and ileo-ileal (case 5). All patients, except case 1, had progressive cancer disease. Only in one patient (case 4), surgery was required due to recurrence of bowel obstruction secondary to peritoneal carcinomatosis. Mean patency time was 1.5–9 months. and death was related to oncological progression.

In two patients with distal EUS-A the stent migrated spontaneously and required removal due to bowel obstruction.

The first patient was a 65-year-old woman with a history of advanced ovarian adenocarcinoma who underwent radical pelvic surgery with hysterectomy, bilateral-salpingo-oophorectomy and sigmoid resection, colorectal anastomosis and diverting ileostomy. Complete obstruction of the colorectal anastomosis was observed during endoscopic-follow-up. Afterwards, she presented a complete disconnection of the surgical anastomosis, with a closed rectal stump and a blind colonic stump, and no oncological relapse.

**Figure 1:**
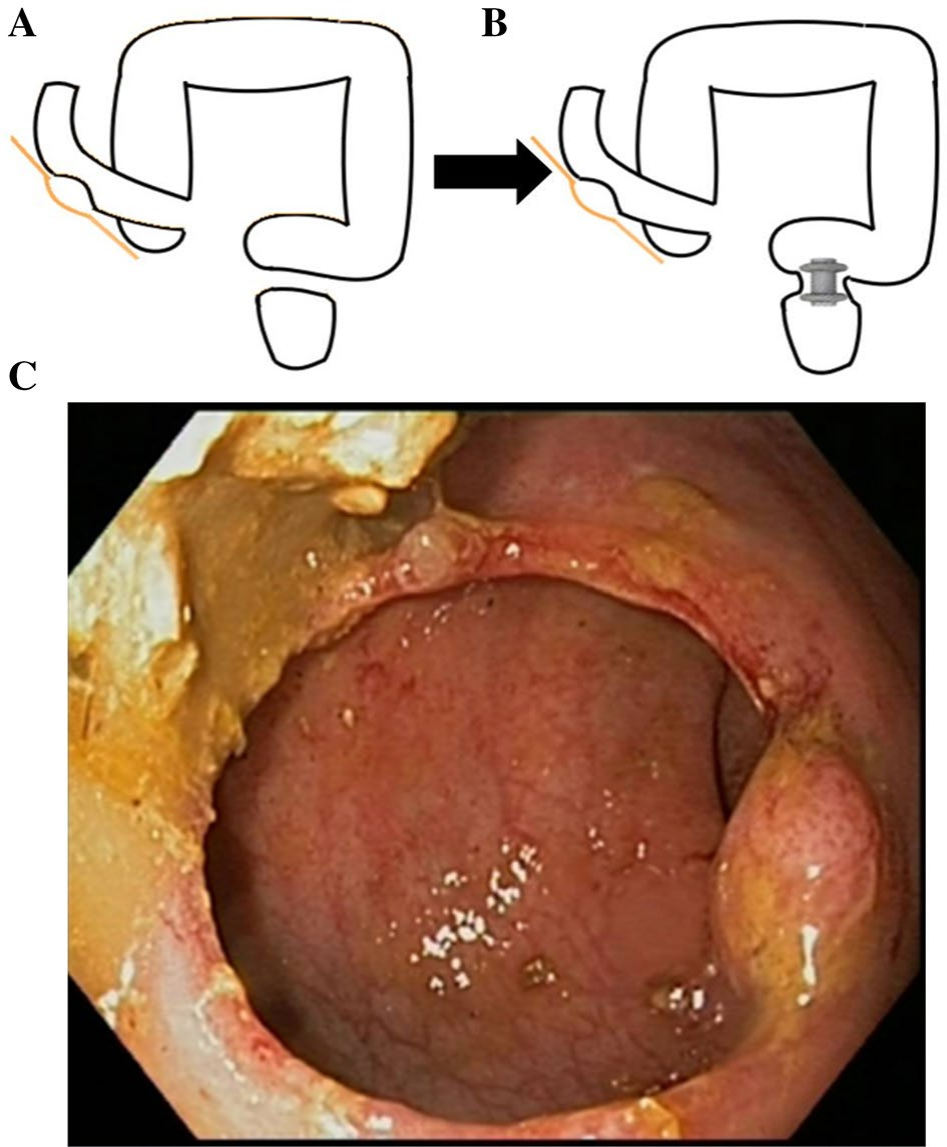
Graphic scheme of the patient's anatomy (**A**, left) and the site of LAMS. placement (**B**, right). (**C**)Endoscopic colorectal anastomosis after removing the LAMS. Currently permeable 12 months after stent removal.

Subsequent treatment options were discussed in a multidisciplinary setting. Surgical treatment was dismissed due to a frozen pelvis and the EUS-guided approach was considered the most appropriate. A pediatric colonoscope was advanced through the ileostomy towards the transverse colon. A guidewire was coiled under fluoroscopic guidance into the colon stump. A nasobiliary drainage catheter was advanced over the wire into the colon, and high-pressure water was injected into the blind segment. The fluid-filled colonic lumen was localized transrectally by EUS and punctured with a cautery-enhanced LAMS (AXIOS-ECTM, 20-mm diameter; Boston Scientific, Galway, Ireland). The proximal flange of the stent was deployed into the colon and the distal flange into the rectal stump. The colorectal endoscopic anastomosis with LAMS was completed without complications (Fig. 1A,B).

Three*-*month follow*-*up endoscopy showed that the stent had migrated distally and confirmed the anastomosis to be large and patent (Fig. 1C). Reversal of the ileostomy was successfully performed. At 15-month follow-up, the patient reported normal bowel movements.

**Figure 2:**
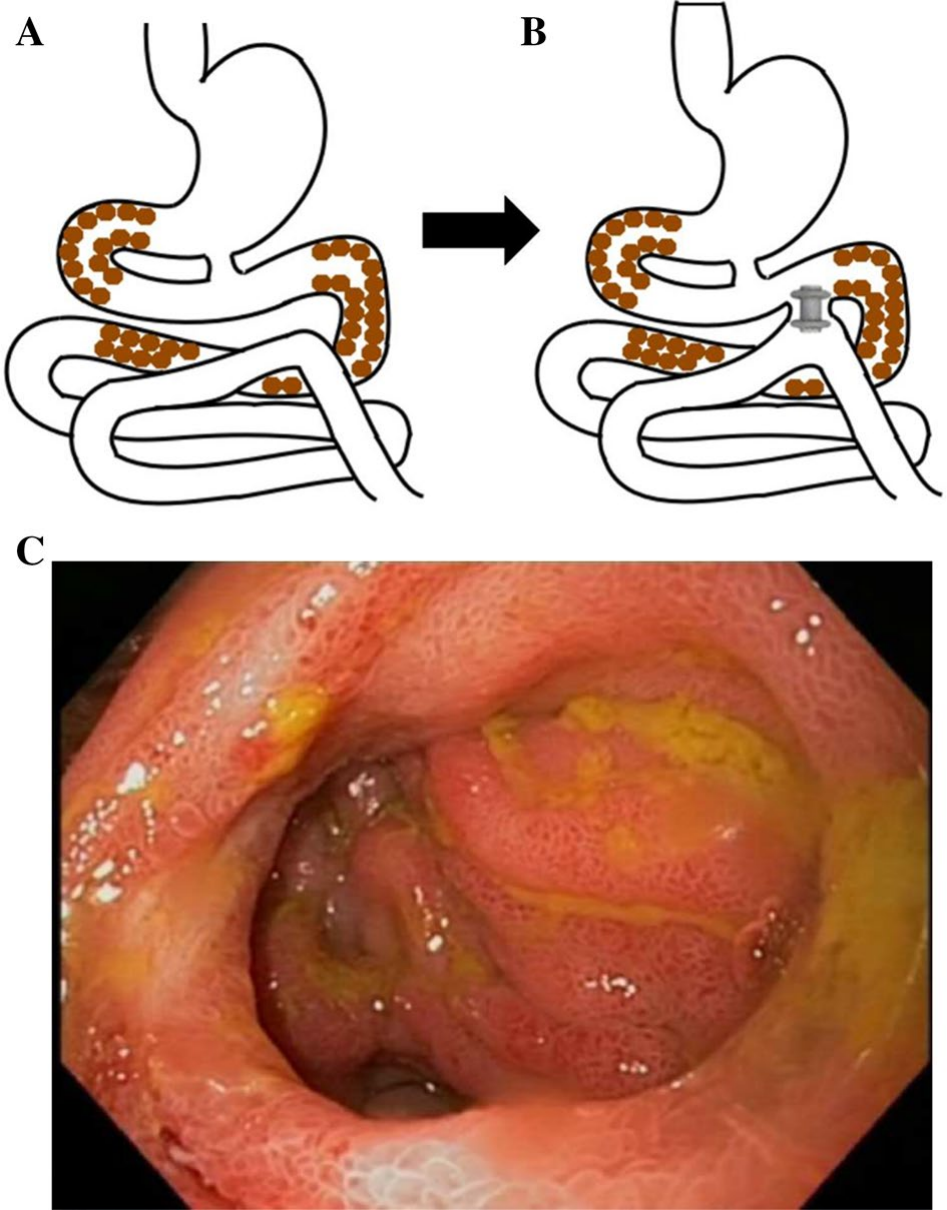
Graphic scheme of the patient's anatomy (**A**, left) and the site of LAMS placement (**B**, right). Brown dots correspond to tumor. (**C**) Jejunal-jejunal anastomosis after removing the LAMS. Subsequent patency: 172 days (until patient death).

The second patient was a 57-year-old man with gastric outlet obstruction due to adenocarcinoma of the anthropyloric region, treated with a palliative surgical gastroenteroanastomosis. Our assessment was requested as the patient complained of repeated vomiting. Gastroscopy showed a complete obstruction of the anthropyloric region. The scope was introduced through the surgical anastomosis in the efferent loop and at 15 cm an infiltrative stenosis was observed. A guide wire was passed deep into the jejunum and, over this wire, an 8.5 F catheter for irrigation with saline and contrast, verifying that the infiltration spread diffusely in the first 30 cm. High-pressure water was infused through the catheter. Subsequently, from the gastric lumen, we looked with the echoendoscope for a distended loop, but all were too far from the gastric wall. Moving towards the stenotic efferent loop through the surgical anastomosis, we identified a small bowel loop, which was distended and close to the prestenotic jejunal wall. A "Hot Axios" LAMS, 20 mm in diameter, was released, creating a lateral-lateral anastomosis between the two intestinal loops (Fig. 2A,B).

The patient progressed well, maintaining oral feeding. Three months later, it was observed that the prosthesis had migrated, occupying the jejunal lumen. As this displacement hindered the passage of food, the stent was retrieved with a foreign body forceps. After stent removal, a permeable and mature lateral-lateral jejunal-jejunal anastomosis was observed (Fig. 2C), so that no further procedures were performed. The patient maintained oral feeding until his death 6 months later.

Given the patency outcome after LAMS migration in these two patients, and after agreeing on the procedure in the Interdisciplinary Committee for Digestive Tumors, we decided to assess the long-term patency of a gastrojejunal anastomosis performed with LAMS after intentional removal of the stent. The patient was a 65-year-old female with Lynch syndrome and GOO due to a duodenal adenocarcinoma; she gave her written informed consent of the off-label LAMS use.

**Figure 3:**
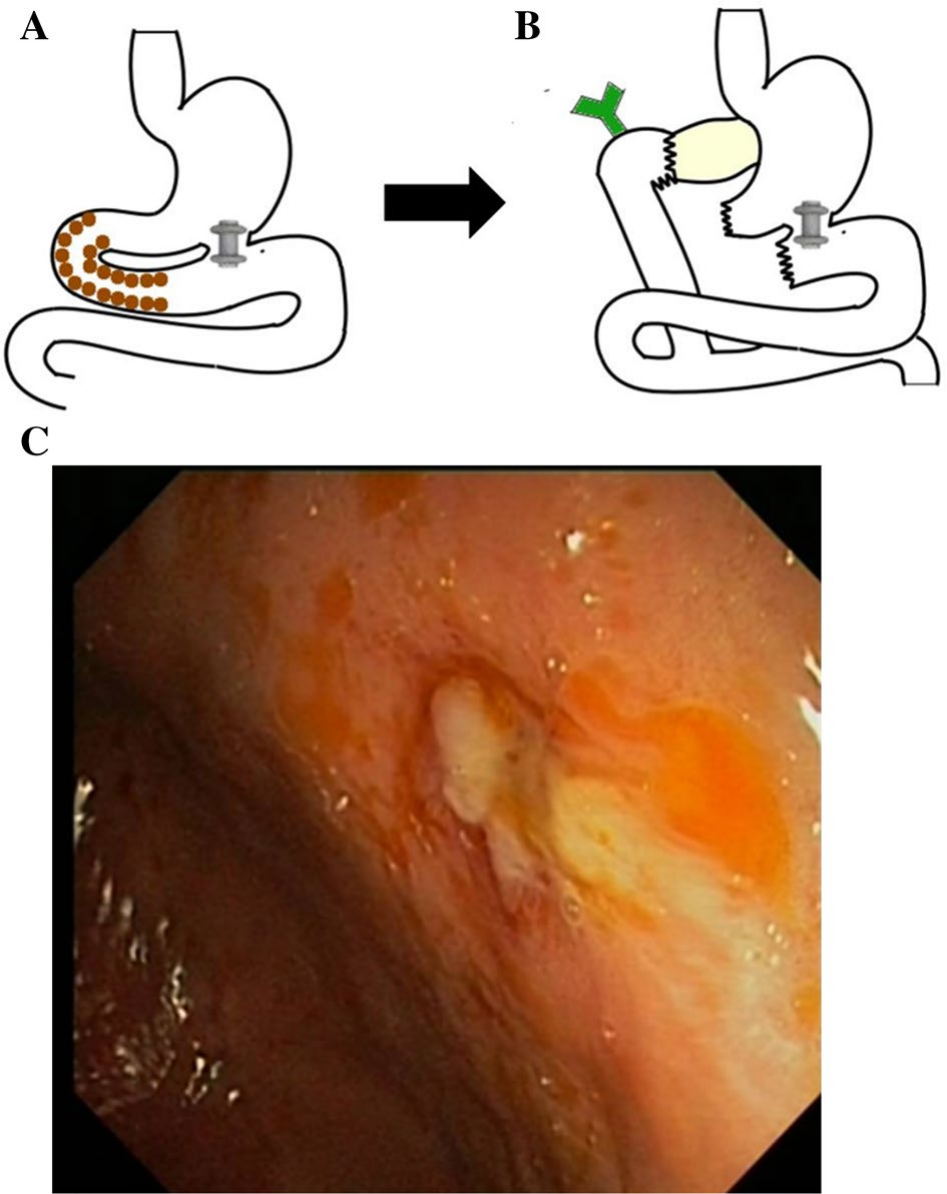
Graphic scheme of the patient's anatomy before (**A**, left) and after Whipple procedure (**B**, right). Brown dots correspond to tumor. (**C**) Gastrojejunal anastomosis 26 days after removal of the LAMS.

She had a history of surgery and chemotherapy for treatment of adenocarcinoma of the right colon and endometrial adenocarcinoma, for which she also received brachytherapy. A gastrojejunal “Hot Axios” was placed. After receiving neoadjuvant chemotherapy for her duodenal carcinoma, the patient was informed of the off-label procedure and gave her written informed consent. The duodenal tumor was resected, and the LAMS was maintained as a permanent anastomosis (Fig. 3A,B) in the context of a Whipple surgery. The stent was removed 4 months after placement, when the fistulous tract was considered mature. Twenty-six days later, the patient presented with symptoms of GOO and the closure of the fistula was confirmed endoscopically (Fig. 3C). A second LAMS was placed and finally a surgical gastroenteroanastomosis was necessary.

## Discussion

Lumen-apposing metal stents (LAMS) were developed for access to and drainage of pancreatic fluid collections ^8,9^. However, LAMS have been increasingly used for other conditions^1^, such as gallbladder drainage, intestinal obstruction, abscess drainage, treatment of afferent loop syndrome, and refractory gastrointestinal strictures^11^. LAMS are fully covered, short and have a wide flange at each end. The short length and wide flanges reduce the risk of migration and improve patient tolerance.

Permanent LAMS placement is not advised in LAMS applications such as pancreatic fluid collection drainage because of the risk of bleeding and stent becoming buried ^12^. Although there is no consensus on whether and when stent removal should be performed, a 4-week interval has been proposed^13^. Little is known about how much time should pass before a LAMS is removed in specific indications such as gastrojejunostomy (EUS-GE), as most studies have focused on short-term outcomes immediately after stent placement^14^. When used for benign GOO, authors recommend removing the stent as soon as there is evidence that the gastric outlet obstruction (GOO) has resolved, via cross-sectional imaging, upper GI series, or endoscopy^4^. In our series, only 3 patients were treated with EUS-GE for benign disease, and two of them are awaiting follow-up. In the largest series reported^4^, LAMS were electively removed in 18 of 21 patients (85.7%) after benign gastric outlet obstruction resolved, with an average dwell time of 270 ± 273 days. The authors do not describe the evolution of the gastroenteric fistula after removal of the LAMS, but it is expected that, once the primary cause of GOO resolves, LAMS removal is followed by progressive closure of the EUS-GE without leaving sequelae. Spontaneous closure of large transmural gastric defects after removal of a AXIOS Stent has been reported^15^. In patients with EUS-directed transgastric ERCP after gastric bypass, follow-up data on fistula closure have been recently reported^16^. All the cases involved the gastric wall, and the authors stated that persistent fistula was uncommon (observed only in 1 of 11 cases). However, these patients presented surgery-altered gastric anatomy (bypass procedure), so these observations may not be applicable to patients with an intact stomach. In our third patient, the gastrojejunal AXIOS stent was retrieved 4 months after deployment, and the anastomosis became completely closed in a few weeks.

We believe that LAMS can be deployed in any part of the gastrointestinal tract, provided that the two walls are close enough and there are no interposed vessels. In addition, the anastomosis site must be accessible to the echoendoscope. Therefore, EUS-A with LAMS between intestinal loops is a procedure reserved for selected patients. In our series, EUS-A did not involve the gastric wall in 6 patients. Data in the literature are scarce and limited to two single cases^8,9^ and afferent loop syndrome^17^. Our experience shows that EUS-A is technically more challenging in these cases, especially if it is necessary to introduce the echoendoscope deeply. There are several problems that make the placement of the LAMS in the distal tract more complex: (1) For transgastric LAMS, the anatomy is a constant since the angle of Treitz is adjacent to the stomach, while in distal anastomoses the anatomy is variable, and this can compromise the passage of the irrigation catheter, the stability of the endoscope and the proximity of the ends to be joined. (2) The method of transgastric anastomosis is quite standardized. However, for distal anastomoses, almost all patients are different, depending on the intestinal segment involved and previous treatments, and technical variations are often necessary. Our main technical failure corresponded to an ileocolic anastomosis. An additional challenge when placing distal (non-gastric) AXIOS stents is the fact that the small bowel is a mobile intraperitoneal organ and can move away during the procedure. Finding the target loop in the small bowel can be difficult, as happened in another of our patients, in whom we placed the LAMS in a jejunal loop instead of placing it into the ileal lumen. Follow up from cases 3, 4, and 5 is limited as patients were deceased within 2 months after LAMS placement. There may be concerns about offering this procedure to patients with a poor prognosis of a few months, but we consider that in these cases, therapeutic EUS offered a palliative approach that provided a better quality of life, allowing oral feeding until death.

Out of our six cases with non-gastric EUS-A, two LAMSs required removal because of migration into the bowel lumen, 117 and 198 days after deployment, respectively. The spontaneously displaced stents were removed endoscopically, and patency of the fistula previously created with LAMS was observed. In the subsequent follow-up, both patients progressed well, and no closure of the anastomosis was observed after 172 days in one case (until the death of the patient) and after 234 days in the other who currently remains asymptomatic. If the long-term patency of EUS-guided anastomosis after the stent retrieval is confirmed in larger series, it can become a minimally invasive approach in complex patients with very few therapeutic alternatives.

We did not achieve long-term permeability of a gastrojejunal anastomosis performed with LAMS after intentional stent removal (4 months after placement). We think that the cause of this different progression once the stent is retrieved or spontaneously migrated, is likely to be related to the variable structure of the *muscularis propria* layer along the digestive tract: in the small intestine, it includes an internal circular layer and an external longitudinal layer; in the colon the longitudinal layer is grouped into three bands called taenia coli, while in the stomach there is a third oblique layer, which can favor the closure of fistulas. Another factor may be that gastric motility^18^ produces a hypertrophic response on the gastric side, with the ability to ingrown foreign bodies, which is not so clearly observed in other segments of the digestive tract. If the gastric wall is involved, it is possible that the use of larger caliber stents^19^ and their later removal could allow the long-term permeability of the anastomosis. However, there are currently no data in the medical literature that support this theory.

Theoretically, LAMS allows adhesion between two organs as is done with a surgical anastomosis^10^. We did not verify if, in those cases in which the patency of the anastomoses was maintained after stent removal, there is a fusion of the different layers of the intestinal wall as was shown in animal models^20^, since one of the patients is currently alive (without clinical obstruction after 4 months) and we have no autopsy data from the deceased patient.

In conclusion, our observations suggest an increased role for EUS-A in the management of patients not only with GOO but also with other different disorders, such us small bowel obstruction (e.g., anastomotic strictures) or non-stentable obstructive neoplasia of the right colon. Distal stenting is more challenging compared to LAMS placement involving the gastric wall, but feasible in some selected cases. Moreover, when the gastric wall is not involved in EUS-A, long-term permeability of the newly created anastomosis may be expected after stent retrieval or spontaneously migrated. In these cases, EUS-A offers the potential benefits of a permanent surgical bypass while maintaining a minimally invasive approach. In the future, well-designed RCTs and prospective studies are needed to further validate these findings.
